# A comparative study of cultural methods for the detection of *Salmonella *in feed and feed ingredients

**DOI:** 10.1186/1746-6148-5-6

**Published:** 2009-02-03

**Authors:** Sevinc Koyuncu, Per Haggblom

**Affiliations:** 1 Department of Chemistry, Environment and Feed Hygiene, National Veterinary Institute, SE-751 89 Uppsala, Sweden

## Abstract

**Background:**

Animal feed as a source of infection to food producing animals is much debated. In order to increase our present knowledge about possible feed transmission it is important to know that the present isolation methods for *Salmonella *are reliable also for feed materials.

In a comparative study the ability of the standard method used for isolation of *Salmonella *in feed in the Nordic countries, the NMKL71 method (Nordic Committee on Food Analysis) was compared to the Modified Semisolid Rappaport Vassiliadis method (MSRV) and the international standard method (EN ISO 6579:2002). Five different feed materials were investigated, namely wheat grain, soybean meal, rape seed meal, palm kernel meal, pellets of pig feed and also scrapings from a feed mill elevator. Four different levels of the *Salmonella *serotypes S. Typhimurium, S. Cubana and S. Yoruba were added to each feed material, respectively. For all methods pre-enrichment in Buffered Peptone Water (BPW) were carried out followed by enrichments in the different selective media and finally plating on selective agar media.

**Results:**

The results obtained with all three methods showed no differences in detection levels, with an accuracy and sensitivity of 65% and 56%, respectively. However, Müller-Kauffmann tetrathionate-novobiocin broth (MKTTn), performed less well due to many false-negative results on Brilliant Green agar (BGA) plates. Compared to other feed materials palm kernel meal showed a higher detection level with all serotypes and methods tested.

**Conclusion:**

The results of this study showed that the accuracy, sensitivity and specificity of the investigated cultural methods were equivalent. However, the detection levels for different feed and feed ingredients varied considerably.

## Background

*Salmonella enterica *contamination of foodstuffs is a considerable human health problem with more than 170 000 human cases reported in the EU in 2005[1]. It is well known that *Salmonella *contamination of animal feed can be disseminated to food producing animals and further down the food chain, causing big economic losses and most importantly be a threat to animal and public health [2,3]. The presence of *Salmonella *in animal feed and feed ingredients is not unusual [1] and cases of human illnesses have been reported where the source of infection were found to be contaminated animal feed [4]. Investigations by the US Food and Drug Administration (FDA) showed that protein-based animal feed are frequently contaminated with *Salmonella enterica *[2]. A major outbreak of human salmonellosis in the United States, United Kingdom, Israel and the Netherlands back in 1970, caused by the, at the time, uncommon serotype *S*. Agona, was traced back to Peruvian fish meal used as an ingredient in the feed manufacturing [2]. Since then *S*. Agona has caused considerable human illness every year in United States [5]. In a study by Davies et al. [6], a strong link between *Salmonella *contamination in feed mills and infections in chickens that received feed from the contaminated feed mills could be established. Sauli et al. [7] pointed out that contaminated animal feed is a significant source of infections in pigs. In 2003, an outbreak of *S*. Cubana in Sweden occurred in a large number of pig farms where the source of infection was traced back to a feed mill that produced pelleted pig feed, indicating the potential effects of feed contamination for further dissemination of *Salmonella *in the food chain [8]. It was found that the source of infection was a contaminated cooler for the pelleted feed where multiplication of *Salmonella *occurred in the humid and warm coatings inside the cooler.

*Salmonella *control of food producing animals has a long history in Sweden and started already in the late 1950s [9]. The present Swedish control program for feed, based on hazard analysis of critical control point (HACCP) principals in the feed mills, was initiated in 1991 by the feed industry [10,11]. Scrapings and dust samples from critical control points (CCPs) in the processing line are analysed for *Salmonella *on a weekly basis giving a rapid alert if *Salmonella *is detected. Imported feed raw materials are sampled for salmonella according to a sampling programme and must remain in quarantine until the analytical results are completed. *Salmonella *positive ingredients are treated with organic acids and re-tested for *Salmonella *before they can be used in the feed manufacturing.

The reported prevalence of *Salmonella *in different feed materials varies considerably between different countries which is most likely due to differences in the sampling and isolation methods used [1]. As pointed out by Gardner [12], lack of comparability may induce bias in the risk estimates if the results from different studies are treated as if the tests and sampling schemes were the same. Animal feed and feed raw materials are usually dry products with a low water activity and the *Salmonella *cells present are strongly dehydrated. For that reason isolation methods for *Salmonella *in feed must be able to regenerate the multiplication of dehydrated and stressed bacterial cells. Geue and Schluter [13] showed that five-fold fractional enrichment of feed and faeces samples resulted in an increased number of *Salmonella *isolations. Validation studies of isolation methods are mainly focused on food and only very few feed materials are usually included in the studies. In a study by Salomonsson *et al*. [14] the levels of competing microflora in different feed materials including scrapings were found in the range of 10^2^–10^7^/g.

Animal feed are produced in large quantities, usually as bulk materials in a batch wise production in feed mills. The numbers of *Salmonella *cells in feed are usually low and their distribution may not be even. According to Maciorowski *et al*. [3] it is crucial to give injured and stressed *Salmonella *cells a chance to recover and multiply in the enrichment in order to successfully isolate *Salmonella *from animal feed.

The international standard method for detection of *Salmonella*, EN ISO 6579:2002, consists of non-selective pre-enrichment in Buffered Peptone Water (BPW), selective enrichment in Rappaport-Vassiliadis with soy broth (RVS) and Müller-Kauffmann tetrathionate-novobiocin broth (MKTTn), plating on the selective solid medium Xylose Lysine Deoxycholat agar (XLD) and a second selective solid medium such as Brilliant Green agar (BGA) and a final serological and biochemical confirmation [15].

The same procedure, without the MKTTn step, is used in the NMKL71 method, which is the standard method for *Salmonella *detection in the Nordic countries for food but also for feed [16].

The Modified Semisolid Rappaport Vassiliadis (MSRV) method, Draft Annex D of EN ISO 6579:2002 [17] is based on migration of motile *Salmonella *through the selective medium [18]. It has been shown in several studies of naturally infected or artificially contaminated food or faecal samples that MSRV is, in most cases, more sensitive than the standard methods [18-21]. However, there are also studies showing that MSRV is less sensitive compared to other enrichment media [19,22]. In the recent EFSA report about microbial risks in feedingstuffs it is recommended that MSRV or alternative methods should be validated also for use in feed [23].

The standard cultural methods are primarily developed for food materials and only very few feed materials have been included in the validations studies. For a bibliography of isolation methods for foodborne *Salmonella *see Schonenbrucher *et al*. [24]. Despite this, methods developed for food are the standard methods used for most feed materials. The levels of *Salmonella *in feed are usually low and an estimate of accuracy based on high spiking levels is thus not relevant [25] and it is important to validate the methods at realistic levels before they are adopted as official standard methods for feed.

The aim of the present study was to evaluate the NMKL71, MSRV and the EN ISO 6579:2002 methods in terms of their relative ability to detect low levels of *Salmonella *in artificially contaminated feed and feed ingredients and also to investigate the accuracy, sensitivity and specificity of respective method. To our knowledge, no such comparisons have so far been performed with feed materials. It was also of interest to find out if there were any differences in detection levels of some serotypes often found in feed materials.

## Results

**Figure 1:**
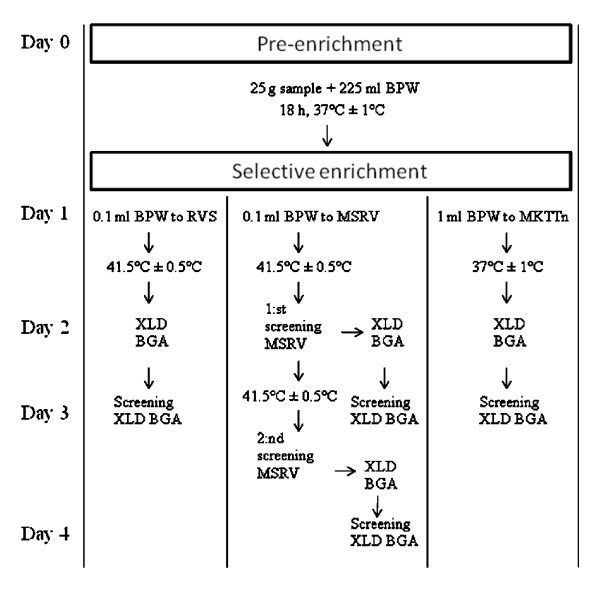
**Flow chart diagram for isolation of *Salmonella *with the NMKL71 (RVS), MSRV or EN ISO 6579:2002 methods (RVS + MKTTn)**. (see methods).

In Figure 1 a flow-chart diagram of the different steps of respective method are presented. For all methods 3–4 days are required for isolation of *Salmonella*. The a_w _of the feed materials used in the study were < 0.66 before spiking and after spiking < 0.68. All non-spiked feed materials were negative for *Salmonella *with the NMKL71 method, which was also later confirmed by the EN ISO 6579:2002 and MSRV methods.

All three methods compared in the present study managed to detect *Salmonella *with an average accuracy and sensitivity of 65% or 56% respectively, with only very small differences between the methods (Table 1). Selective enrichment in RVS, MRSV or in MKTTn resulted in identical detection levels on XLD medium. The different serotypes added were detected at similar detection levels for all methods (Table 2, 3, 4). In rape seed meal, the serotypes were detected at the spiking level of 1–10 cfu/25 g while for palm kernel meal the detection level was between 10-10^3 ^cfu/25 g. For wheat grain and scrapings the detection levels of the serotypes were between 1–10^3 ^cfu/25 g materials. The detection levels for the different feed materials are summarized in Table 5.

**Table 1:** Performance of NMKL71, MSRV and EN ISO 6579:2002 in feed materials.

	**NMKL71**	**MSRV**	**EN ISO 6579:2002**
TP*	190	190	191
TN*	85	85	85
FP*	0	0	0
FN*	150	150	149

AC**	0.65	0.65	0.65
SE**	0.56	0.56	0.56
SP**	1	1	1

*TP = True positives, TN = True negatives, FP = False positives, FN = False negatives.

**AC = Accuracy, SE = Sensitivity, SP = Specificity (As described in materials and methods).

Positive or negative results were biochemically and serologically confirmed and shown as total numbers for each method.

**Table 2:** Detection of *Salmonella *Typhimurium in artificially contaminated animal feeds by the NMKL71 (RVS), MSRV and EN ISO 6579:2002 methods (RVS + MKTTn).

Feed type	Selective medium	cfu/25 g of *Salmonella *Typhimurium					
		
		0	0–1	1–10	10-10^2^	10^2^–10^3^	10^3^–10^4^
Pellets of pig feed	RVS	0/5	0/5	0/5	5/5	5/5	-*
	MSRV	0/5	0/5	0/5	5/5	5/5	-
	MKTTn	0/5	0/5	0/5	5/5	5/5	-

Soybean meal	RVS	0/5	0/5	5/5	5/5	5/5	-
	MSRV	0/5	0/5	5/5	5/5	5/5	-
	MKTTn	0/5	0/5	5/5	5/5	5/5	-

Palm kernel meal	RVS	0/5	-	0/5	0/5	5/5	5/5
	MSRV	0/5	-	0/5	0/5	5/5	5/5
	MKTTn	0/5	-	0/5	0/5	5/5	5/5

Rape seed meal	RVS	0/5	0/5	5/5	5/5	5/5	-
	MSRV	0/5	0/5	5/5	5/5	5/5	-
	MKTTn	0/5	0/5	5/5^d^	5/5^d^	5/5	-

Wheat grain	RVS	0/5	0/5	5/5	5/5	5/5	-
	MSRV	0/5	0/5	5/5	5/5	5/5	-
	MKTTn	0/5	0/5	5/5	5/5^c^	5/5^b^	-

Scrapings from feed mill	RVS	0/5	0/5	0/5	5/5	5/5^a^	-
	MSRV	0/5	0/5	0/5	5/5	5/5	-
	MKTTn	0/5	0/5	0/5	5/5^d^	5/5^d^	-

* Not tested.

a = One negative BGA plate.

b = Two negative BGA plates.

c = Three negative BGA plates.

d = Five negative BGA plates.

**Table 3:** Detection of *Salmonella *Cubana in artificially contaminated animal feeds by the NMKL71 (RVS), MSRV and EN ISO 6579:2002 methods (RVS + MKTTn).

Feed type	Selective medium	cfu/25 g of *Salmonella *Cubana				
		
		0	0–1	1–10	10-10^2^	10^2^–10^3^
Pellets of pig feed	RVS	0/5	0/5	0/5	5/5	5/5
	MSRV	0/5	0/5	0/5	5/5	5/5
	MKTTn	0/5	0/5	0/5	5/5^d^	5/5

Soybean meal	RVS	0/5	0/5	0/5	5/5	5/5
	MSRV	0/5	0/5	0/5	5/5	5/5
	MKTTn	0/5	0/5	0/5	5/5^a^	5/5

Palm kernel meal	RVS	0/5	0/5	0/5	5/5	5/5
	MSRV	0/5	0/5	0/5	5/5	5/5
	MKTTn	0/5	0/5	0/5	5/5^b^	5/5

Rape seed meal	RVS	0/5	0/5	5/5	5/5	5/5
	MSRV	0/5	0/5	5/5	5/5	5/5
	MKTTn	0/5	0/5	5/5^d^	5/5^e^	5/5^e^

Wheat grain	RVS	0/5	0/5	0/5	5/5	5/5
	MSRV	0/5	0/5	0/5	5/5	5/5
	MKTTn	0/5	0/5	0/5	5/5	5/5

Scrapings from feed mill	RVS	0/5	0/5	5/5^d^	5/5^c^	5/5^a^
	MSRV	0/5	0/5	5/5	5/5	5/5
	MKTTn	0/5	0/5	5/5^e^	5/5^e^	5/5^e^

a = One negative BGA plate.

b = Two negative BGA plates.

c = Three negative BGA plates.

d = Four negative BGA plates.

e = Five negative BGA plates.

**Table 4:** Detection of *Salmonella *Yoruba in artificially contaminated animal feeds by the NMKL71 (RVS), MSRV and EN ISO 6579:2002 methods (RVS + MKTTn).

Feed type	Selective medium	cfu/25 g of *Salmonella *Yoruba				
		
		0	0–1	1–10	10-10^2^	10^2^–10^3^
Pellets of pig feed	RVS	0/5	0/5	0/5	5/5	5/5
	MSRV	0/5	0/5	0/5	5/5	5/5
	MKTTn	0/5	0/5	0/5	5/5	5/5

Soybean meal	RVS	0/5	0/5	5/5	5/5	5/5
	MSRV	0/5	0/5	5/5	5/5	5/5
	MKTTn	0/5	0/5	5/5	5/5	5/5

Palm kernel meal	RVS	0/5	0/5	0/5	0/5	5/5
	MSRV	0/5	0/5	0/5	0/5	5/5
	MKTTn	0/5	0/5	0/5	0/5	5/5^b^

Rape seed meal	RVS	0/5	0/5	5/5	5/5	5/5
	MSRV	0/5	0/5	5/5	5/5	5/5
	MKTTn	0/5	0/5	5/5^d^	3/5^c^	5/5^c^

Wheat grain	RVS	0/5	0/5	0/5	0/5	5/5
	MSRV	0/5	0/5	0/5	0/5	5/5
	MKTTn	0/5	0/5	0/5	1/5^a^	5/5

Scrapings from feed mill	RVS	0/5	0/5	0/5	0/5	5/5
	MSRV	0/5	0/5	0/5	0/5	5/5^a^
	MKTTn	0/5	0/5	0/5	0/5	5/5^e^

a = One negative BGA plate.

b = Two negative BGA plates.

c = Three negative BGA plates.

d = One BGA and one XLD plate negative.

e = Five negative BGA plates.

**Table 5:** Detection levels obtained with NMKL71, MSRV and EN ISO 6579:2002 for different feed materials and *Salmonella *serotypes (cfu/25 g).

	*S*. Typhimurium	*S*. Cubana	*S*. Yoruba
Pellets of pig feed	10-10^2^	10-10^2^	10-10^2^
Soybean meal	1–10	10-10^2^	1–10
Palm kernel meal	10^2^–10^3^	10-10^2^	10^2^–10^3^
Rape seed meal	1–10	1–10	1–10
Wheat grain	1–10	10-10^2^	10^2^–10^3 ^*
Scrapings from feed mill	10-10^2^	1–10	10^2^–10^3^

*One XLD plate plated from MKTTn at 10-10^2 ^cfu/25 g was detected positive.

The sensitivity and specificity for the methods at different spiking levels are summarized in Table 6. No false positives were recorded, indicating a specificity of > 97% for each of the methods. Since the detection level in palm kernel meal was markedly different this feed material was excluded from Table 6. For palm kernel meal the sensitivity at spiking levels 10–100 cfu and 100–1000 cfu was in the range 14–57% for all three methods. At the level 1000–10000 cfu the sensitivity was in the range 60–100% for all three methods. The wide confidence intervals are due to the small sample size.

**Table 6:** Sensitivity and specificity of the NMKL71 (RVS), MSRV and EN ISO 6579:2002 (RVS + MKTTn) methods at different spiking levels of Salmonella.

Selective medium	Specificity (%)	Sensitivity (%)			
	
	cfu/25 g of *Salmonella*				
	
	0	0–1	1–10	10-10^2^	10^2^–10^3^
RVS	> 97	< 4	36–58	78–93	> 96
MSRV	> 97	< 4	36–58	78–93	> 96
MKTTn	> 97	< 4	36–58	76–92	> 96

The reported range defines a 95% confidence interval for the sensitivity and specificity respectively. Palm kernel meal was excluded from the calculation.

The detection level was slightly different in each feed type and the lowest spiking level of 0–1 cfu/25 g did not generate any *Salmonella *positive samples with any of the serotypes (Tables 2, 3, 4). One XLD plate from MKTTn was positive at 10-10^2 ^cfu/25 g for wheat spiked with *S*. Yoruba that was not detected by the other selective media. MKTTn failed to detect *Salmonella *in two out of five replicates at 10-10^2 ^cfu/25 g for rape seed meal spiked with *S*. Yoruba.

The RVS in comparison with the MKTTn medium produced fewer false negative isolates on BGA plates (Tables 2, 3, 4). The XLD and BGA plates were more contaminated with competing microflora from the enrichment in MKTTn broth than from the RVS broth. The intrinsic flora was more abundant on XLD and BGA plates from scrapings compared to other feed materials tested and selective enrichment of scrapings in MKTTn, plated on BGA plates, did not generate any typical *Salmonella *colonies in any experiment.

On the MSRV plates no additional positive results, after incubation for another 24 h, was observed. The XLD and BGA plates plated from MSRV contained considerably less intrinsic flora compared to plates from MKTTn or RVS broth. Serological agglutination of isolated colonies confirmed the presence of added serotypes.

The average pH values of the different feed and feed ingredients, measured after the pre-enrichment, are shown in Table 7. The pH values were between 4.9 and 6.5 with rape seed meal showing the lowest and wheat grain the highest value.

**Table 7:** Average pH values measured after pre-enrichment (BPW) of the feed and feed ingredients at 37°C for 18 h (2–4 measurements).

Feed type	pH value
Pellets of pig feed	5.0
Soybean meal	5.2
Palm kernel meal	6.0
Rape seed meal	4.9
Wheat grain	6.5
Scrapings from feed mill	5.0

## Discussion

The objective of the present study was to investigate the relative performance of common cultural methods for *Salmonella *in animal feed and feed ingredients using artificially contaminated samples. By stressing the *Salmonella *bacteria before isolation attempts were made to simulate the more natural conditions prevailing in feed materials. Because of the low levels of *Salmonella *potentially present in animal feed, low levels of *Salmonella *were added to the feed samples and for that reason the calculated accuracy and sensitivity data are lower than reported in other studies [20].

The volume of the bacterial cultures added to the respective feed material did not generate any significant change of the a_w_, clearly indicating that available moisture did not allow any bacterial growth before the BPW was added. *Salmonella *was not detected in any of the non-spiked samples by any method and serotyping of isolated strains verified that no other than the added *Salmonella *serotypes were present in the feed materials.

The detection levels observed with MSRV, MKTTn or RVS were similar with the feed materials tested. However, the detection levels varied for the different feed and feed ingredients investigated and for palm kernel meal it was higher for all serotypes compared to the other feed materials tested. At the lowest spiking level, stochastic events will result in an uneven distribution of cells between individual samples, and it is possible that some samples contained no or only few cells. This fact may have contributed to the low sensitivity observed at these spiking levels. The reasons why low levels of *Salmonella *are not detected in palm kernel meal is presently unknown. However, the most likely explanation might be poor growth of *Salmonella *during the enrichment in the presence of palm kernel meal. The higher detection levels in palm kernel meal might leave *Salmonella *positive consignments undetected when screening of feed ingredients are carried out.

According to several studies, a low pH value during the pre-enrichment might affect the viability of *Salmonella *bacteria [19,26] and according to the EN ISO 6579:2002 method the pH value should stay above 4.5. In the present study the lowest pH-value was detected for rape seed meal (4.9). For palm kernel meal, with the highest detection level of *Salmonella*, the pH-value was 6.0, indicating no correlation between a low pH value and a low isolation rate. Other factors that might affect the re-isolation of *Salmonella *could be the presence of potential growth inhibitors in the feed materials or competing bacterial flora.

Positive BGA plates were more difficult to interpret when inoculated from MKTTn, most likely because of high levels of competing microflora and few characteristic *Salmonella *colonies. D'Aoust [27] showed that members of *Citrobacter*, *Enterobacter*, *Escherichia *and *Proteus *could be resistant to tetrathionate, resulting in reduced selectivity for the MKTTn broth.

Salomonsson *et al*. [14] showed that wheat and scrapings contained high numbers of aerobic microorganisms and also *Escherichia coli*. According to Van Schothorst and Renaud [28] a high level of intrinsic flora in the sample, after enrichment, may reduce isolation of *Salmonella *bacteria on BGA. A comparison between XLD plates and BGA-plates showed that XLD is superior to BGA, due to higher selectivity [22,29], which is in agreement with the results in the present study. Re-incubation of MSRV plates for an additional 24 h did not increase the number of positive samples and an uneven halo was typically observed, not representing growth of *Salmonella*.

The cultural methods compared in this study are primarily developed to detect *Salmonella *in different food samples and since the level of intrinsic flora is usually high in animal feed [14] the isolation of *Salmonella *from feed might be affected. Another drawback is that the cultural methods are time consuming which might delay possible control measures. MSRV is unable to detect non-motile *Salmonella *bacteria, that represent < 1.0% of the isolates from animal feeds [30]. However, this study shows that selective enrichment on MSRV results in lower level of competing flora on the XLD and BGA plates then does enrichment in other media.

## Conclusion

In conclusion, the cultural methods in the present study were shown to be surprisingly equivalent in terms of accuracy, sensitivity and specificity for different feed materials and serotypes, respectively. An interesting observation was the differences in detection levels between different feed and feed ingredients.

## Methods

NMKL71:5, the international standard method EN ISO 6579:2002 and the Draft Annex D of EN ISO 6579:2002, using the MSRV as a selective enrichment medium, were compared, in relation to their accuracy, specificity and sensitivity for different feed ingredients and feed materials.

### Feed materials

Four feed ingredients (wheat grain, soybean meal, rape seed meal, palm kernel meal), finished feed (pellets of pig feed) and scrapings from elevator were collected from a Swedish feed mill and stored at 4–8°C until used. A representative sample of all feed materials were analysed with the NMKL71 method before the experiments started. For each feed material the pH value was measured in the buffered peptone water (BPW) (Oxoid CM 0509, Basingstoke, England) after the pre-enrichment. The water activity (a_w_) of the feed and feed ingredients was determined at ambient temperature before and after addition of bacterial culture. AquaLab Series 3 analyzer (ADAB Analytical Devices AB, Stockholm) was used according to the manufacturer's instructions.

### Salmonella strains

*Salmonella enterica *ssp. *enterica *serotype Typhimurium ST115506 (*S*. Typhimurium), *S*. Cubana ST58403 and *S*. Yoruba ST45506 (from the culture collection at the National Veterinary Institute, Sweden), all isolated from animal feed, were used in the experiments. The strains were stored at -70°C and were cultured on brom-cresol-purpure-lactose agar plates (blue-agar) (Oxoid). On day 1, one colony from the plate was inoculated in 5 ml serum broth (National Veterinary Institute, Sweden) and incubated at 37°C for 18–24 h. Ten-fold dilution series in peptone saline water were made on day 2. The number of cfu in the peptone saline water (PSW) was determined by plating out 0.1 ml from selected dilutions on blue-agar plates, which were incubated at 37°C overnight. In order to simulate more natural conditions, where *Salmonella *might be stressed, the bacterial cells were kept at 4–8°C in PSW for two days and then re-counted before the experiments were carried out.

### Sample preparation and pre-enrichment

The NMKL71, MSRV and the EN ISO 6579:2002 methods were run in parallel and in each experiment one *Salmonella *serotype, three feed materials and a non-spiked sample of each feed were included. Twenty-five grams of each feed material was weighed into a plastic jar and spiked with 0–1, 1–10, 10-10^2 ^or 10^2^–10^3 ^cfu, except for the palm kernel meal spiked with *S*. Typhimurium, which received ten times higher levels (1–10, 10-10^2^, 10^2^–10^3 ^or 10^3^–10^4 ^cfu/25 g). The volumes used for spiking were approx. 370 μl/25 g. The samples were left in room temperature for 4 h before 225 g of BPW was added, followed by incubation at 37° ± 1°C for 18 h.

### Selective enrichment and confirmation

Three drops (equivalent to approximately 0.1 ml) of the BPW were placed separately and equally spaced on the surface of Modified Semisolid Rappaport Vassiliadis agar plates (MSRV) (Oxoid CM 0910) supplemented with 1.0% Novobiocin (Sigma-Aldrich N1628) and then incubated in an upright position due to its semi-solid composition at 41.5° ± 0.5°C for 24 ± 3 h. In parallel, three drops of BPW were inoculated in 10 ml Rappaport-Vassiliadis broth (RVS) (Oxoid CM 0866) and 1 ml to 9 ml Müller-Kauffmann tetrathionate-novobiocin broth (MKTTn) (Oxoid CM 0343). The RVS broth was incubated in a water bath at 41.5° ± 0.5°C for 24 ± 3 h, and the MKTTn at 37° ± 1°C for 24 ± 3 h. Five replicates of each level were used.

After incubation the MSRV plates were examined for suspect *Salmonella *growth and a sample was plated on Xylose Lysine Deoxycholat agar (XLD) (Lab M lab 32, Axel Johnson Lab System Inc. Solna, Sweden) (with 1.5% Novobiocin) and Brilliant Green agar (BGA) (Oxoid CM 0329). If no migration was noted the plates were incubated for additional 24 h at 41.5° ± 0.5°C and the same procedure was applied. A loop (0.1 ml) of each MKTTn and RVS- broth was also plated on XLD and BGA and incubated at 37° ± 1°C for 24 ± 3 h and then screened for *Salmonella *colonies.

Five colonies, for each XLD/BGA plate from the lowest spiking level, were plated on blue-agar plates and incubated at 37° ± 1°C for 24 ± 3 h. Confirmation was done by serological agglutination with monovalent anti-O sera, O-13 and O-23 to detect *S*. Cubana, O-4 and O-5 for *S*. Typhimurium and O-16 for *S*. Yoruba. Physiological NaCl was used in order to check for auto-agglutination.

### Statistical analysis of performance criteria

Since the spiking levels for palm kernel meal with *S*. Typhimurium were higher than for the other feed materials, those data were excluded from the calculation of overall accuracy, sensitivity and specificity. The detection level of each method for the different animal feed materials spiked with the respective *Salmonella *serotype was compared. The accuracy (AC), sensitivity (SE) and specificity (SP) were calculated for each method and statistical analysis was done according to the principals used by European Commission Directorate General Joint Research Centre [31]. This approach was recently used by Eriksson and Aspán [20] in order to evaluate isolation methods for *Salmonella *in faeces. The assumption is that all non-spiked samples are negative for *Salmonella *and only those samples spiked with *Salmonella *are true positives. Samples being positive on at least one selective agar plate, that is XLD, BGA or both, are considered positive. Based on this, the accuracy, sensitivity and specificity rates were obtained by using the following definitions and equations [20]:

TP   True Positives

A sample was defined as true positive when *Salmonella *was detected in a sample where *Salmonella *had been added

TN   True Negatives

A sample was defined as true negative when *Salmonella *was not detected in a sample where *Salmonella *had not been added

FP   False Positives

A sample was defined as false positive when *Salmonella *was detected in a sample where *Salmonella *had not been added

FN   False Negatives

A sample was defined as false negative when *Salmonella *was not detected in a sample where *Salmonella *had been added

Accuracy (AC) = (TP+TN)/(TP+TN+FP+FN)

Accuracy (AC) is a measure for the ability of a method to correctly classify samples containing *Salmonella *as positive for *Salmonella*, and samples not containing *Salmonella *as negative for *Salmonella*.

Sensitivity (SE) = TP/(TP+FN)

Sensitivity (SE) is a measure for the ability of a method to classify a sample containing *Salmonella *as positive for *Salmonella*.

Specificity (SP) = TN/(TN+FP)

Specificity (SP) is a measure for the ability of a method to classify a sample not containing *Salmonella *as negative for *Salmonella*.

In the present study the relative difference in accuracy, sensitivity and specificity between the different methods were investigated.

When accuracy and specificity are investigated at different spiking levels and in different feed materials the sample number are small and the estimated performance measures are associated with a great deal of uncertainty. To account for this the respective accuracy, sensitivity and specificity under these conditions are reported as Bayesian confidence intervals [32], rather than point estimates.

The accuracy and specificity of the tests are reported as shortest confidence intervals [33], under the assumption that all values are equally probable. The calculations were performed using an online calculator on (, Dec 16, 2008). The values reported defines the boundaries of an interval that, with 95% certainty, contains the true value of the accuracy, sensitivity or specificity.

## Authors' contributions

SK outlined the design, performed the laboratory procedures and the statistical analysis, and drafted the manuscript. Interpretation of the results was performed by both authors. PH coordinated the study and helped to draft the manuscript. Both authors read and approved the final manuscript.

## References

[B1] (2006). The community summary report on trends and sources of zoonoses, zoonotic agents, antimicrobial resistance and foodborne outbreaks in the European Union in the 2005. The EFSA Journal.

[B2] Crump JA, Griffin PM, Angulo FJ (2002). Bacterial contamination of animal feed and its relationship to human foodborne illness. Clin Infect Dis.

[B3] Maciorowski KG, Herrera P, Jones FT, Pillai SD, Ricke SC (2006). Cultural and Immunological Detection Methods for Salmonella spp. in Animal Feeds – A Review. Vet Res Commun.

[B4] Hald T, Wingstrand A, Brondsted T, Lo Fo Wong DM (2006). Human health impact of Salmonella contamination in imported soybean products: a semiquantitative risk assessment. Foodborne Pathog Dis.

[B5] Helfrick DL, Olsen SJ, Bishop RD (2000). An atlas of Salmonella in the United States: serotype-specific surveillance, 1968–1998.

[B6] Davies R, Breslin M, Corry JE, Hudson W, Allen VM (2001). Observations on the distribution and control of Salmonella species in two integrated broiler companies. Vet Rec.

[B7] Sauli I, Danuser J, Geeraerd AH, Van Impe JF, Rufenacht J, Bissig-Choisat B, Wenk C, Stark KD (2005). Estimating the probability and level of contamination with Salmonella of feed for finishing pigs produced in Switzerland – the impact of the production pathway. Int J Food Microbiol.

[B8] Osterberg J, Vagsholm I, Boqvist S, Lewerin SS (2006). Feed-borne outbreak of Salmonella Cubana in Swedish pig farms: risk factors and factors affecting the restriction period in infected farms. Acta Vet Scand.

[B9] Karlsson K-A, Rutqvist L, Thal E (1963). Salmonella isolated from Animals and Animal Feeds in Sweden during 1958–1962. Nord Vet Med.

[B10] Haggblom P (1993). Monitoring and control of Salmonella in animal feed. International course on Salmonella control in animal production and products 1993; Malmö, Sweden.

[B11] Sternberg Lewerin S, Boquist B, Engström P, Häggblom P, Mead GC (2005). The effective control of Salmonella in swedish poultry. Food safety in the poultry industry.

[B12] Gardner IA (2004). An epidemiologic critique of current microbial risk assessment practices: the importance of prevalence and test accuracy data. J Food Prot.

[B13] Geue L, Schluter H (1998). A Salmonella monitoring programme in egg production farms in Germany. Zentralbl Veterinarmed B.

[B14] Salomonsson AC, Aspán A, Johansson S, Heino A, Häggblom P (2005). Salmonella detection by polymerase chain reaction after pre-enrichment of feed samples. Journal of Rapid Methods and Automation in Microbiology.

[B15] (2002). Microbiology of food and animal feeding stuffs – Horizontal method for the detection of Salmonella spp. ISO 6579:2002 E standard 1, rue de Varembé, CH 1211.

[B16] (1999). NMKL (Nordic Committee on Food Analysis) Salmonella detection in foods.

[B17] Detection of Salmonella spp. in animal faeces and in samples from the primary production stage. Draft Amendment ISO 6579: 2002/amendedDAmd 1, (2006-09-12) Amendment 1 Annex D.

[B18] De Smedt JM, Bolderdijk R, Rappold H, Lautenschlaeger D (1986). Rapid Salmonella detection in foods by motility enrichment on modified semisolid Rappaport-Vassiliadis medium. Journal of Food Protection.

[B19] De Zutter L, De Smedt JM, Abrams R, Beckers H, Catteau M, de Borchgrave J, Debevere J, Hoekstra J, Jonkers F, Lenges J (1991). Collaborative study on the use of motility enrichment on modified semisolid Rappaport-Vassiliadis medium for the detection of Salmonella from foods. Int J Food Microbiol.

[B20] Eriksson E, Aspan A (2007). Comparison of culture, ELISA and PCR techniques for salmonella detection in faecal samples for cattle, pig and poultry. BMC Vet Res.

[B21] Voogt N, Raes M, Wannet WJ, Henken AM, Giessen AW van de (2001). Comparison of selective enrichment media for the detection of Salmonella in poultry faeces. Lett Appl Microbiol.

[B22] Wiberg C, Norberg P (1996). Comparison between a cultural procedure using Rappaport-Vassiliadis broth and motility enrichments on modified semisolid Rappaport-Vassiliadis medium for Salmonella detection from food and feed. Int J Food Microbiol.

[B23] (2008). Scientific Opinion of the Panel on Biological Hazards on a request from the Health and Consumer Protection, Directorate General, European Commission on Microbiological Risk Assessment in feedingstuffs for food producing animals. The EFSA Journal.

[B24] Schonenbrucher V, Mallinson ET, Bulte M (2008). A comparison of standard cultural methods for the detection of foodborne Salmonella species including three new chromogenic plating media. Int J Food Microbiol.

[B25] Glaeser H (2004). Detection of pathogenic micro-organisms-a contribution to discussion. Dtsch Tierarztl Wochenschr.

[B26] van Schothorst M, Renaud AM (1985). Malachite green pre-enrichment medium for improved salmonella isolation from heavily contaminated samples. J Appl Bacteriol.

[B27] D'Aoust JY (1989). Foodborne Bacterial Pathogens, Salmonella. Doyle MP edn New York.

[B28] van Schothorst M, Renaud AM (1983). Dynamics of salmonella isolation with modified Rappaport's medium (R10). J Appl Bacteriol.

[B29] Bauwens L, Vercammen F, Bertrand S, Collard JM, De Ceuster S (2006). Isolation of Salmonella from environmental samples collected in the reptile department of Antwerp Zoo using different selective methods. J Appl Microbiol.

[B30] Poppe C, Mann ED, Shaw S, Warburton D, Sewell A (2004). Procedure for the isolation of Salmonella species by the modified semi-solid Rappaport Vassiliadis (MSRV) method.

[B31] Boix A, Von Holst C, Baeten V, Berben G, Vancutsem J (2004). Determination of Processed Animal Protein (PAPS) including meat and bone meal (MBM) in feed. Part I and II.

[B32] Jaynes ET, Harper WL, Hooker CA (1976). Confidence Intervals vs. Bayesian Intervals. Foundations of Probability Theory, Statistical Inference, and Statistical Theories of Science.

[B33] Nicholson BJ (1985). On the F-Distribution for Calculating Bayes Credible Intervals for Fraction Nonconforming. IEEE Transactions on Reliability.

